# Why There Are Exactly Two Sexes

**DOI:** 10.1007/s10508-025-03348-3

**Published:** 2025-11-04

**Authors:** Colin M. Wright

**Affiliations:** The Manhattan Institute for Policy Research, 52 Vanderbilt Avenue, New York, NY 10017 USA

Across anisogamous species, the existence of two—and only two—sexes has been a settled matter in modern biology (Lehtonen et al., 2016; Parker et al., 1972). “Male” designates organisms whose biological function is to produce small gametes (sperm), and “female” designates organisms whose biological function is to produce large gametes (ova) (Hilton & Wright, 2023; Minot, 1888; Smith, 1978). This nomenclature reflects two divergent reproductive strategies that recur across a wide range of taxa (Togashi & Cox, 2011). As with the fact of evolution itself, contemporary scientific debates have long moved on from questioning whether the sex binary is a fact to questions about how anisogamy evolved, why it persists, and what its evolutionary consequences are.

In recent years, however, this previously uncontroversial fact has been challenged in popular discourse (Fuentes, 2023; Kralick, 2018; Viloria & Nieto, 2020) and now increasingly in scholarly scientific publications (Ainsworth, 2015; Fuentes, 2025; McLaughlin et al., 2023; Velocci, 2024), seemingly driven by cultural and political debates surrounding the concept of “gender identity” and transgender rights. Popular outlets now routinely publish articles asserting that there are more than two sexes or that sex is a nonbinary “spectrum” conceived as a continuum or as a multivariate cluster of traits. Scholarly articles have amplified this framing by characterizing the sex binary as overly simplistic, outdated, and even oppressive, urging its replacement with broader and putatively more nuanced models (Ainsworth, 2015).

Here I synthesize evolutionary and developmental evidence to demonstrate that sex is binary (i.e., there are only two sexes) in all anisogamous species and that males and females are defined universally by the type of gamete they have the biological function to produce—not by karyotypes, secondary sexual characteristics, or other correlates.

I begin with a concise account of sexual reproduction and the evolution of anisogamy, explaining why two differentiated gamete types evolved from isogamous ancestors and how this functional dimorphism grounds the categories “male” and “female.” I then assess five recurrent arguments used to undermine the binary nature of sex: (1) conflating “mating types” with sexes; (2) treating sex chromosome aneuploidies and karyotype variation as additional sexes; (3) reclassifying differences/disorders of sex development as evidence for a nonbinary “sex spectrum”; (4) recasting sex as a polythetic cluster of traits; and (5) proposing “multilevel” models that attach sex labels not to individual organisms but only to specific traits. Finally, I outline the scientific and societal benefits of definitional clarity, and close by explaining why attempts to decenter gametes are inherently self-defeating because all coherent routes to “male” and “female” remain intelligible only with respect to sperm and ova.

## The Evolutionary and Biological Basis of the Sex Binary

### What Sex Is and Why It Evolved

Sexual reproduction is the formation of a new organism by the fusion of haploid gametes (syngamy) produced via meiosis (Alberts et al., 2014). By reshuffling genetic variation through recombination and outcrossing, sexual reproduction generates novel genotypes and can reduce the genetic load, which helps explain its persistence despite the costs (Kondrashov, 1988; Otto, 2009).

At the gametic level, sexual reproduction occurs in two broad modes that differ in the size of gametes being fused (Togashi & Cox, 2011). In isogamy, fusion involves gametes of equal size. In anisogamy, fusion involves differently sized gametes (Lehtonen et al., 2016). Anisogamy is thought to have evolved over one billion years ago (Butterfield, 2000) from isogamous ancestors via disruptive selection favoring two distinct gamete types: small, typically motile gametes optimized for quantity and encounter rate (sperm) and large, nutrient-rich gametes optimized for provisioning (ova) (Levitan, 2006; Parker, 2011; Parker et al., 1972). Working in concert, these divergent strategies maximize fertilization success and offspring survival.

The sexes—male and female—refer to these two distinct reproductive strategies in anisogamous species. Males are defined as the sex that produces numerous small gametes (sperm). Females, conversely, are defined as the sex that yields fewer but larger gametes (ova) (Parker, 2011; Williams, 1975). Accordingly, individuals are categorized as male or female based on whether their reproductive system has the biological function to produce sperm or ova, respectively (Bogardus, 2025). Because sperm and ova are the only two gamete classes in anisogamous systems, there are only two sexes. This gametic dimorphism underlies biologists’ reference to sex as a “binary” (Hilton & Wright, 2023).

## Common Arguments Against the Binary and Why They Fail

### Sexes as Mating Types

Popular and scholarly articles sometimes claim that certain fungi and slime molds have “hundreds” or even “thousands” of sexes (GrrlScientist, 2019; Scharping, 2017). These claims commit a category error by conflating mating types with *sexes* (Lehtonen et al., 2012). In isogamous taxa, mating types are genetically or molecularly defined compatibility classes that regulate which gametes can fuse; they are not differentiated by gamete size (Hurst & Hamilton, 1992). By contrast, sexes in anisogamous taxa are defined by gametic dimorphism—the production of small gametes (sperm) versus large gametes (ova). Some anisogamous species may also possess mating-type systems layered on top of male and female functions, but isogamous species, by definition, lack sexes.

Claims of hundreds or thousands of sexes thus refer to many mating types in isogamous systems, not to sexes. Where reproduction is anisogamous, the number of sexes remains two—male and female—defined by gamete type (Lehtonen, 2021).

### Sexes as Karyotypes

A common line of argumentation used to undermine the binary nature of sex does so by misrepresenting the sex binary as a binary of human karyotypes (XX = female; XY = male) and then citing sex chromosome aneuploidies (e.g., XXY in Klinefelter syndrome; X0 in Turner syndrome) or rare XX males and XY females to claim there must be “more than two sexes” (Ainsworth, 2015; Blackless et al., 2000). How could sex be binary and determined by sex chromosomes, they argue, if there are more viable sex chromosome karyotypes in humans beyond XX and XY?

The fundamental flaw is conflating how sex is determined with how it is defined (Capel, 2017; Griffiths, 2021; Hilton & Wright, 2023). In developmental biology, sex determination refers to the mechanisms that trigger and regulate sexual development. These mechanisms vary widely across taxa (Bachtrog, 2014). Examples include chromosomal (e.g., SRY gene on Y chromosome in mammals), temperature-dependent (e.g., higher temperatures produce males in many reptiles), haplodiploidy (e.g., unfertilized haploid eggs yield males in most Hymenoptera insects), or environmental (e.g., chemical cues in *Bonellia viridis*).

Yet, regardless of the mechanism by which sex is determined, an individual’s sex—male or female—is universally defined by the type of gamete (sperm or ova) their reproductive system has the biological function to produce (Goymann et al., 2023). Sex chromosome aneuploidies therefore represent variations within the two sexes, not additional sexes.

### Sex as a Spectrum

While claims that there are “more than two sexes” are common, the most frequent challenge against the binary nature of sex asserts that sex is a “spectrum” (Ainsworth, 2015; Fuentes, 2025). On this view, “male” and “female” are not distinct biological categories, but theoretical endpoints of a continuous distribution that individuals can only ever statistically approximate. This model entails that individuals can only be described in degrees of maleness and femaleness, rather than strictly male or female. The primary evidence invoked to support the spectrum model is the existence of disorders/differences in sex development (DSDs) (Sax, 2002), including forms of genital or gonadal atypicality, often presented visually along a continuum from “typical female” to “typical male.”

However, the existence of such conditions does not undermine the binary nature of sex, because the sex binary does not entail that every individual can be unambiguously categorized as male or female. Rather, the claim is that in anisogamous organisms there are only two gamete types, sperm and ova, and thus only two sexes. Sexual ambiguity is not a third or intermediate sex because developmental variation does not correspond to producing new gamete types.

### Sexes as Polythetic Categories

A polythetic category is one in which members share overlapping characteristics, with no single feature necessary or sufficient for membership. Inclusion is based on “family resemblance”: Each member shares enough traits with others to be recognized as part of the set, even though not all members share the same combination of traits (Needham, 1975; Wittgenstein, 1953).

Proponents of a polythetic sex model draw on this idea to portray sex as multivariate (rather than univariate, as in a simple “spectrum”). On this view, “sex” is an aggregate of traits—chromosomes, gonads, gametes, hormones, neuroanatomy, secondary sex characteristics, and other sexually dimorphic traits—and individuals are assigned degrees of maleness or femaleness according to how their overall profile aligns with what is considered male-typical or female-typical (Dreger, 2000; Fausto-Sterling, 2000).

However, male and female are not polythetic categories. They are reproductive classes defined by a single criterion: The type of gamete (sperm or ova) an organism’s reproductive system has the biological function to produce. All other traits—karyotype, genital morphology, hormone profiles, neurological and somatic dimorphisms—are typically causes, proxies, or consequences of that functional distinction. Treating those correlates as jointly definitional blurs the determinants and downstream effects of sex with sex itself.

Moreover, the polythetic approach is logically self-refuting (Griffiths, 2021). Traits are labeled “male-typical” or “female-typical” *because* they correlate with males and females already identified independently—ultimately by reference to gametes. In other words, the model presupposes the binary categories rooted in gametes it seeks to replace and then infers those categories back from their correlates. As a descriptive framework for trait variation, a multivariate summary can be useful; as a definition of sex, it is nonsensical.

### The Multilevel Sex Model

The multilevel model blends spectrum and polythetic approaches by distributing “sex” across several putative “levels”, commonly including sex chromosomes, internal reproductive anatomy, external genitalia, secondary sexual characteristics, hormone profiles, and behavior, and sometimes extending to “gender identity” (Migeon & Wisniewski, 1998). Rather than classifying organisms as male or female, the model assigns sex labels to traits or levels (e.g., “genetic sex,” “endocrine sex,” “morphological sex,” “behavioral sex,” and so on) (McLaughlin et al., 2023; Sun et al., 2023).

As articulated by McLaughlin et al. (2023), sex is framed as “a constructed category operating at multiple biological levels,” with four focal levels: genetic, endocrine, morphological, and behavioral. This framing conflates the determinants and correlates of sex with sex itself (Bachtrog, 2014; Capel, 2017). Genes and gene networks initiate and regulate sexual differentiation; hormones mediate downstream development and phenotypic dimorphisms; morphology and many behaviors are influenced by an organism’s sex. Yet none of these traits defines sex. Sex is an organism-level reproductive class anchored to the type of gamete that organism has the biological function to produce. Treating upstream regulators (e.g., SRY activity, hormonal milieu) or downstream outcomes (e.g., dimorphic morphology, behavior) as coequal “levels” of sex is a level-of-analysis error.

Moreover, the multilevel account inherits the same circularity as the polythetic model. Traits are labeled “male-typical” or “female-typical” only because they correlate with organisms already identified as male or female—an identification that, in anisogamous species, is made ultimately by reference to gametes. Once that reference is removed, the typology loses its interpretive footing. As a descriptive framework to integrate genetic, endocrine, and morphological findings in clinical differential diagnosis, the multilevel schema has pragmatic value; as a definition of sex, it is incoherent.

## Conclusion

This commentary advances a simple claim with broad consequences: In anisogamous organisms, the sexes—male and female—are functional classes defined by the type of gamete an individual has the biological function to produce (Bogardus, 2025). Males have the biological function to produce sperm; females have the biological function to produce ova (Parker et al., 1972). That definition is universal across all anisogamous taxa. Much contemporary confusion arises from conflating how sex is determined (i.e., how sex develops) with how sex is defined (what sex is), and from conflating upstream determinant and downstream correlates of sex with sex itself. On this account, aneuploidies and DSDs describe variation in development or function *within* the two sexes; “mating types” belong to isogamous systems and are compatibility classes, not sexes; and “multivariate” or “spectrum” depictions quantify trait variation within and between the two sexes without altering the number of sexes.

The scientific value of clear and precise definitions is enormous (Dawkins, 2025). A gamete-based definition prevents error propagation across comparative biology, physiology, ecology, and medicine. It preserves the interpretability of sex-linked phenomena—sexual selection, dimorphism, and life-history trade-offs—and maintains conceptual discipline by keeping determination mechanisms (e.g., SRY pathways, ZW systems, temperature-dependent determination, social cues) in their proper explanatory lane. It also secures cross-taxon coherence: Whether a species is gonochoric or hermaphroditic, and whether determination is chromosomal, environmental, or social, “male” and “female” remain meaningfully comparable because those terms are anchored to reproductive function rather than to a bundle of traits that shift widely from taxa to taxa.

The societal and ethical stakes are also significant. Accurate biology is distinct from questions of dignity, rights, and how we treat one another. Policy disputes should not be adjudicated by redefining—or defining away—the reproductive realities that make sex a useful scientific concept in the first place. When categories are blurred for nonscientific reasons, we invite downstream harms: muddled clinical protocols, compromised epidemiology, eroding and/or conflicting legal protections, and diminished public trust in science.

Across anisogamous taxa, males and females are defined by gametic dimorphism. Proposals to redefine sex in terms of karyotypes, secondary sexual characteristics, behavior, or other correlates are incoherent and invariably presuppose this foundation, because the categories “male” and “female” are intelligible only by reference to sperm and ova.
